# Substrate-Induced Response in Biogas Process Performance and Microbial Community Relates Back to Inoculum Source

**DOI:** 10.3390/microorganisms6030080

**Published:** 2018-08-05

**Authors:** Tong Liu, Li Sun, Åke Nordberg, Anna Schnürer

**Affiliations:** 1 Department of Molecular Science, Swedish University of Agricultural Sciences, Uppsala 75007, Sweden; tong.liu@slu.se (T.L.); li.sun@slu.se (L.S.); 2 Department of Energy and Technology, Swedish University of Agricultural Sciences, Uppsala 75007, Sweden; ake.nordberg@slu.se

**Keywords:** anaerobic digestion, co-digestion, continuous stirred-tank reactor (CSTR), bio-methane potential (BMP)-test, next-generation amplicon sequencing, terminal restriction fragment length polymorphism (T-RFLP), qPCR, glycoside hydrolase families 5 and 48

## Abstract

This study investigated whether biogas reactor performance, including microbial community development, in response to a change in substrate composition is influenced by initial inoculum source. For the study, reactors previously operated with the same grass–manure mixture for more than 120 days and started with two different inocula were used. These reactors initially showed great differences depending on inoculum source, but eventually showed similar performance and overall microbial community structure. At the start of the present experiment, the substrate was complemented with milled feed wheat, added all at once or divided into two portions. The starting hypothesis was that process performance depends on initial inoculum source and microbial diversity, and thus that reactor performance is influenced by the feeding regime. In response to the substrate change, all reactors showed increases and decreases in volumetric and specific methane production, respectively. However, specific methane yield and development of the microbial community showed differences related to the initial inoculum source, confirming the hypothesis. However, the different feeding regimes had only minor effects on process performance and overall community structure, but still induced differences in the cellulose-degrading community and in cellulose degradation.

## 1. Introduction

Biogas, produced via anaerobic digestion, represents a valuable renewable energy resource that can replace part of the fossil fuel-based energy used today, resulting in climate and economic benefits [1]. Many types of organic materials can be used for biogas production, but agricultural residues (manure and crop residues, such as stalks, straw, husks, cobs, grass, etc.) are of particular interest due to high abundance and thus high gas potential [2]. However, the high content of lignocellulose and nutrient imbalances often limit the degradation efficiency of agricultural residues [3]. An additional limitation with manure is high water content, making it difficult to achieve high organic loads and volumetric gas production [4]. Some of the obstacles with these types of materials can be overcome by various pre-treatment methods, making the material more accessible to microbial and enzymatic attack [5], or by co-digestion with materials that provide complementary nutrients [6]. For manure-based biogas plants, co-digestion also offers possibilities to increase the organic load. By combining manure with a high-water content with a more energy-dense material, such as crop/crop residues, the organic load can be increased without significantly decreasing the hydraulic retention time (HRT) [4,6,7].

A prerequisite to achieving efficient biogas production is an active microbial community in balance [8]. Parameters shown to impact the community include operating parameters, such as temperature, organic loading rate (OLR), substrate composition, and feeding regime [9,10,11]. Many studies have looked for correlations between microbial composition and reactor function, but most have not found consistent relationships [12,13]. Some studies suggest positive correlations between diversity and function [14,15], but a correlation between low diversity and high function has also been reported [16]. Still, positive correlations are often seen in connection with a specific type of substrate/environment. For example, for the function of processes operating with protein-rich materials and consequently high ammonia concentrations, the importance of syntrophic acetate-oxidizing bacteria has been highlighted [17]. For the degradation of lipids, positive correlations with the level of *Syntrophomonas* have been shown [18]. For cellulose degradation, positive correlations with the level of *Clostridium cellulolyticum* have been observed [12]. Feeding regime has also been shown to influence function, diversity, and community structure [9,19,20,21,22,23], with some studies showing positive correlations between more frequent feeding regime and higher microbial diversity [19,21,23], however, not necessarily resulting in better reactor function [9,22,23,24].

The aim of the present study was to increase the understanding of the relationship between community structure and performance and efficiency of a biogas process operating with lignocellulose-rich substrates. More specifically, the aim was to investigate the importance of original inoculum source, used during start-up, when adding a cosubstrate to biogas processes operated with the same substrate and showing similarity in both performance and overall microbial composition. The hypothesis is that the reactors will respond differently to the change in substrate depending on the microbial community present in the initial inoculum.

To test the hypothesis, reactors operated in a previous study were used in the present experimental work [15]. These reactors were initially started with different inocula characterized by differences in community structure and diversity, and fed a mixture of cow manure and silage grass. The reactors initially showed significant differences in degradation efficiency and methane yield but over time, after operation for more than 3 HRT, the processes became similar regarding both performance and overall community structure and diversity, as analyzed by targeting 16S rRNA gene [15]. However, specific analysis of the potential cellulose-degrading community, targeting the genes encoding *cel 5* and *48* glycosidases, revealed that the reactors still differed in this regard at the end of the experiment [15]. In the present study, these reactors were complemented with milled feed wheat (MFW) as an additional cosubstrate. It was selected as a cosubstrate because its high total solids (TS) concentration allowed the organic load to be increased without significantly altering the HRT. The MFW was either added all at once, together with the grass-manure mixture, or divided into two portions, in order to evaluate also the effect of feeding regime. The reactors were operated for more than 3 HRT and their overall performance regarding methane yield stability and changes in microbial community structure were investigated. Both the total microbial community and potential cellulose degrading bacteria were analyzed. Degradation of the substrates and of pure cellulose was also investigated in batch cultures started with inoculum from the reactors at the beginning and end of the experimental period.

## 2. Materials and Methods

### 2.1. Laboratory-Scale Semi-Continuous Anaerobic Reactors

**Figure 1:**
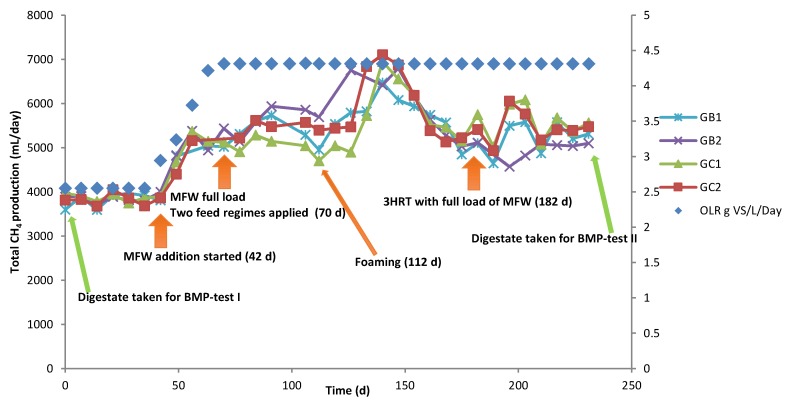
Primary Y-axis: total average methane (CH_4_) production (mL/day) in four continuous laboratory-scale biogas reactors originally started with two different types of inoculum (GB, GC) and codigested with substrates of grass–manure and milled feed wheat (MFW) in two feeding regimes, full load (GB1, GC1) and split load (GB2, GC2) at 37 °C. The methane values were standardized to normal atmospheric pressure (273.15 K, 1 bar). Secondary Y-axis: the total OLR (g VS of the substrates per L reactor volume per day).

Two laboratory-scale continuous stirred-tank reactor (CSTR) processes in duplicate reactors were initially started with inoculum from two different full-scale biogas processes (codes GB, GC) in Sweden [15]. Operating information on the full-scale plants can be found in our previous publications [12,15]. Based on the inoculum origin, the reactors were named GB1 and GB2, and GC1 and GC2. In the 120 days before the start of the present experiment (day 0), the reactors were fed with the same substrate, a grass-manure mixture (Table 1) [15], for six days a week (once a day), with an average daily load of 2.6 g volatile solids (VS)/L and 40-day HRT. After 42 days of operation in the present study, MFW (Table 1) was added to all four reactors with average daily load gradually increasing from 0.6 to 1.7 g VS/L (6 days a week, from day 42 to 70) (Figure 1), resulting in a total average daily load of 4.3 g VS/L and a HRT of 37 days. From day 70, the reactors were fed with the full load of MFW (1.7 g VS/L day) in different feeding regimes: reactors GB1 and GC1 were fed all MFW at the same time as the grass-manure mixture, while GB2 and GC2 were fed the MFW in two portions, with half the amount fed 2 h after adding the grass-manure mixture and the remaining half after another 2 h. In total, the reactors were operated for 231 days, corresponding to 4.5 HRT at a full load of MFW. All reactors were operated at mesophilic (37 °C) temperature, a stirring speed of 90 rpm, and a HRT of 37 days. Samples of liquid (15 mL) were taken at day 0, 77, 106, 147, and 231 and frozen at −20 °C for later analysis of the microbial community structure. 

### 2.2. Anaerobic Batch Test

The methane potential of the substrate was analyzed by a bio-methane potential (BMP) test [15], performed on two time points. On the first time point (test I), digestate samples from the duplicate reactors, before MFW addition, were pooled and used as inoculum (GB0_0 and GC0_0). On the second time point (test II), inocula were taken from all the laboratory-scale reactors after 231 days of operation, corresponding to an operating period of 4.5 HRT at full MFW load, and used in separate tests (GB1_231, GB2_231, GC1_231, and GC2_231). The grass-manure mixture, MFW, and cellulose (control group) were evaluated on both time points, i.e., with all inocula. Before starting the tests, all inocula were kept at 37 °C for seven days to decrease biogas production from the endogenous material. At the start of the BMP test, inoculum and substrate were mixed in a serum bottle (309 mL) under flushing with nitrogen gas (N_2_). The amount of inoculum and substrate was 12 g and 3 g VS/L, respectively, i.e., the inoculum to substrate ratio was 4:1 [25,26]. Tap water was added to the bottles to reach a final liquid volume of 193 mL. Each substrate was evaluated in triplicate bottles. Additionally, to monitor background gas production from inoculum alone, three bottles were initiated by adding the same amount of inoculum and water to reach the same final liquid volume, but with no substrate. All bottles were incubated on a rotary shaker at 37 °C and 130 rpm. Gas production was quantified by pressure measurements, and the methane content was analyzed by sampling (2 mL) followed by analysis by gas chromatography (GC) [27]. After each sampling, the pressure in the bottles was released. The biogas and methane values were standardized to normal atmospheric pressure (273.15 K, 1 bar). The accumulated amount of methane was plotted over time, and the value obtained after leveling off was considered the specific methane production (ml CH_4_/g VS). The efficiency of methane production in each reactor was further evaluated by transferring the time (days) spent to reach 50%, 80%, and 100% of the final methane potential.

### 2.3. Residual Methane Emissions Measurement

The residual methane potential of the digestate was measured twice, on the same time points as the BMP tests, by incubating 50 mL digestate from the four laboratory-scale reactors, i.e., digestate from day 0 and day 231, at 37 °C for approximately 52 days, when the methane production leveled off. Sampling and analysis of methane production during the incubation were performed according to the method described above.

### 2.4. Analytical Methods

2 mL gas samples were withdrawn weekly from each reactor to determine the methane content by gas chromatography according to a previously described method [27]. At the same time, 15 mL liquid samples (digestate) were taken to determine pH [28] and volatile fatty acid (VFA) content by high-performance liquid chromatography (HPLC) [27]. The pH was determined directly after sampling. Liquid samples (400 mL) were also taken on two-time points and sent to a commercial analytical laboratory (Agrilab, Uppsala, Sweden) for determination of the concentration of total nitrogen and ammonium-nitrogen according to standard ISO methods 13878:1998 and 11732:2005, respectively. Total carbon was measured according to standard ISO 10694 and total phosphorus, total sulfur, and total potassium according to Swedish standard SS 28311. Composition of the MFW was determined by Nord Mills Co. and that of the grass-manure mixture by the laboratory at the Department of Animal Nutrition and Management (Swedish University of Agricultural Sciences, Uppsala, Sweden). Starch content was determined by an enzymatic method according to Åman and Hesselman (1984). Crude protein content was analyzed according to the Nordic Committee on Food Analysis (1976) method for nitrogen determination in food and feed (Kjeldahl, No 6, 3rd Edn), using a 2520 Digestor, Kjeltec 8400 Analyzer unit, and 8460 sample unit (FOSS Analytical A/S Hilleröd, Denmark). Crude fat was determined according to the Official Journal of the European Communities method for determination of crude oils and fat (Commission Directive 98/64/EC, 1998), using a Hydrotec 8000 and Soxtec 8000 extraction unit (FOSS Analytical A/S Hilleröd, Denmark). Weight of total solids and volatile solids in the inocula and substrate samples was measured according to international standard methods published by the American Public Health Association (1998).

### 2.5. DNA Extraction and Microbial Community Analysis

For DNA extraction, liquid samples (15 mL) from the semi-continuous processes were taken at the time for starting the present study (inocula, 0 day) and after 77, 106, 147, and 231 days (around 1, 2, 3, and 5 HRT, respectively) of operation with the MFW and the grass–manure mixture. Aliquots of 200 mg in triplicate were used to extract total genomic DNA, as described previously [15]. The degenerate primer sets 515’F and 805R were used to amplify the 16S rRNA genes of both archaea and bacteria to build amplicon libraries for next-generation amplicon sequencing [29]. The PCR products were purified by using AMPure XP (Beckman Coulter, Inc. Brea, CA, USA) and eluted with 20 μL EB buffer, and quantified by using Qubit (Invitrogen, Thermo Fisher Science, Waltham, MA, USA). The concentrations of the final PCR product were adjusted to 5 nM with EB buffer and 2 μL of each final adjusted PCR product were pooled together. The next-generation amplicon sequencing was performed at SciLifeLab in Uppsala, Sweden, using MiSeq system. The raw DNA sequencing data obtained were submitted to the National Center for Biotechnology Information database (NCBI) under accession number: from SRR5808389 to SRR5808384, and analyzed through the open-source bioinformatics pipeline: Quantitative Insights into Microbial Ecology (QIIME) with loaded module bioinfo-tools, QIIME/1.8.0/1.9.1, SeqPrep and Cutadapt [17]. Specifically, the adaptor and primer sequences were trimmed using the following criteria: (1) Trim base from the 3′ end which had a quality below 10. (2) Remove read if it contained N base, was longer than 300 bp, or did not contain primer sequences. The trimmed paired end reads were further processed in QIIME/1.8.0/1.9.1 [30]. Join_paired_ends.py was used for joining paired end reads with minimum overlap 150 bp bases, using the SeqPrep method (https://github.com/jstjohn/SeqPrep). The joined reads were used for splitting into libraries with no barcode errors allowed, and only one consecutive low-quality base call was allowed per read. Any read that with Phred quality scores below 20 were removed. Then, the operational taxonomic units (OTUs) were assigned by using the open reference OTU pick strategy [31]. The criteria for OTU clustering was set to a threshold of 97% similarity and performed with Uclust against Greengenes core set (gg_13_8) [32]. The most abundant sequence in each OTU was selected as a representative sequence and further aligned against the Greengenes core set using PyNAST software [33]. The chimeric sequences were discarded using ChimeraSlayer [34]. Taxonomy was assigned to each OTU using the Ribosomal Database Project (RDP) classifier with a minimum confidence threshold of 80% [35]. Then, OTUs that at least be observed in three samples and contained at least 0.0025% of total reads were retained and used to build the final OTU table. Alpha diversity (Chao1, Shannon and Simpson index and richness) and Beta diversity (unweighted UniFrac distance matrix) analysis were performed using QIIME/1.8.0/1.9.1 [30].

According to the result from sequencing, three methanogenic groups: Methanobacteriales, Methanomicrobiales, and Methanosarcinaceae were quantified by quantitative polymerase chain reaction (qPCR) using the primer sets MBT, MMB, and Msc, respectively [27]. The qPCR protocol and analysis were performed as described previously [27]. The potential cellulose-degrading bacterial community in the substrate and in the digestate after 154 days of operation was analyzed by terminal restriction fragment length polymorphism (T-RFLP) targeting the genes of glycoside hydrolase families 5 and 48, according to the procedure described previously [12]. The length patterns of the fragments obtained were compared with the sequences of clone libraries established in our earlier publications [12,36].

## 3. Results and Discussion

### 3.1. BMP Test

In the first BMP test (test I), the final methane potential of all substrates tested reached a mean value of 357 ± 45 mL CH_4_/g VS and showed no significant difference between the different substrates or the different inocula (pairwise *t*-test, *p* > 0.05) (Table 2). These values were at the same level as observed before for similar substrates, e.g., cow manure, grass, and cellulose [4,12]. The values in the second test were in the same range, but some differences could be seen. The average BMP value for cellulose and MFW reached 291 ± 46 and 300 ± 38 mL CH_4_/g VS, respectively, with a slightly higher value for cellulose in GB1_231 compared with GB2_231 (Table 2 and Figure S1). For the grass–manure mixture the final methane potential in the second BMP test (test II) using inoculum from the GB reactors was significantly lower as compared with test I; 252 ± 29 (GB1_231) and 289 ± 11 (GB2_231) compared to 329 ± 27 mL CH_4_/g VS (GB0_0) (pairwise *t*-test < 0.01). For GC, however, the values for MFW remained more similar to those in test I; 327 ± 46 (GC1_231) and 375 ± 73 (GC2_231) compared to 382 ± 71 mL CH_4_/g VS (GC0_0) (Table 2). Moreover, the average BMP value obtained for the grass–manure mixture in GC1_231 and GC2_231 (353 ± 62 mL CH_4_/g VS) was significantly higher than in GB1_231 and GB2_231 (270 ± 28 mL CH_4_/g VS) (student *t*-test, *p* < 0.02), suggesting the importance of initial inoculum source.

The time to reach the final methane potential was in the first test (I) between 28–52 days, with the longest time for the grass manure mixture. This suggests that the manure grass substrate will not be fully degraded in the semi-continue process having 30 days retention time. Moreover, significantly longer times were needed to reach the final potential, as well as 50% and 80% of this potential, for all the substrates, in the second (II) compared with the first (I) test (student *t*-test, *p* < 0.01) (Table 2). Still, higher degradation efficiency for cellulose was seen in GB1_231 compared with GB2_231, which might indicate a small effect of the different feeding approaches for this process (Table 2 and Figure S1).

### 3.2. CSTR Processes

This experiment was started with four reactors previously operated in another study [15], where they were initially started with two different inocula and were shown to have very different performance in the initial phase of operation, but similar performance by the end. In the present study, the reactors showed similar initial performance after complementing the grass–manure mixture with MFW. Irrespective of the feeding regime, co-digestion with MFW increased the total methane production compared with the grass–manure mixture alone in all four semi-continuous processes. In the initial phase, the level increased gradually from 3818 ± 158 to 5317 ± 304 mL CH_4_/day (average value for day 0–42 and day 56–112, respectively) and then increased rapidly and reached a peak of 6669 ± 439 mL CH_4_/day on day 140. Thereafter, total methane production decreased gradually and stabilized at 5362 ± 205 mL CH_4_/day (day 182–231), i.e., after 3 HRT of operation with a full load of MFW (Figure 1). This final value represented an increase of around 29% over the initial level before addition of the MFW, as also observed in our previous study [15].

As expected, increasing the load by addition of MFW resulted in a significant increase in volumetric gas production, thus giving more efficient use of available digester volume. Several previous studies have shown a similar positive effect of codigesting energy-dense materials with manure [4,6,7,37]. However, an increase in OLR also resulted in foaming (day 112) and increased VFA levels, suggesting some instability in the processes (Table S1). Still, this foaming was only temporary and lasted for approximately two weeks, after which the problem stopped. Shortly after (on day 140) a peak was observed in the CH_4_ production (Figure 1, Figure S2; 312 ± 16 mL CH_4_/g VS). This peak might be explained by nutrients accumulating and being converted to CH_4_ by microorganisms when the foam disappeared.

Even though an increase in total methane yield was obtained, both specific methane production (SMP, defined as the normalized volume of CH_4_ produced per g VS of the substrate) and degree of degradation (VS reduction) decreased in response to MFW addition (Figure S2). The SMP level in all digesters was on average 296 ± 16 mL CH_4_/g VS before addition of MFW (day 0–42) and 249 ± 18 mL CH_4_/g VS after addition (day 182–231). This decrease was not expected as the BMP values for the grass–manure mixture and the MFW were similar and thus the SMP should theoretically not decrease (Table 2). Still, the results were also supported by the BMP tests, illustrating decreased degradation efficiency in test II compared to test I (Table 2). Moreover, the corresponding VS reduction before and after MFW addition was 74.1 ± 3.4% and 63.7 ± 3.5%, respectively. Thus, combined these results suggest that the increase in load by addition of MFW resulted in less efficient degradation than when the grass–manure mixture was used as the sole substrate.

Differences in feeding regime did not result in any statistically significant differences in volumetric or specific methane yield. However, there were some minor differences related to process performance, e.g., VFA accumulation. Accumulation of VFA started around day 112 in all reactors, when the OLR reached 4.3 g VS/L day (Table S1). The total level fluctuated somewhat but was highest at day 224 (1.6–4.6 g/L). Accumulation of VFA is linked to less efficient biogas production and typically occurs when there is an imbalance between different microbial degradation steps. A high propionate to acetate ratio can be taken as an early indicator of a risk of process failure [38]. In this study, the propionate to acetate ratio showed some differences depending on the feeding regime, with GB1 and GC1 showing slightly higher values than GB2 and GC2 after day 203 (Table S1). The VFA accumulation was also associated with the foaming event, but in that case, no differences related to the feeding regime were observed. Foaming can be triggered by many parameters such as the production of surface-active substances, abrupt degassing, viscosity, alkalinity, insufficient mixing, and accumulation of VFAs [39]. In our previous study, the reactors had been operated with the grass–manure mixture for a long time without any VFA accumulation or foaming [15]. Thus, the instability in the present study was clearly caused by the introduction of MFW as a substrate. In comparison with manure, the MFW had higher levels of protein and starch (Table 1). When protein is degraded ammonium-nitrogen is released. In this study, the ammonium-nitrogen concentration increased from 1.05 ± 0.5 to 2.6 ± 0.12 g/L as a result of MFW addition (average of all reactors, from day 0 to 224). Ammonium is in equilibrium with ammonia, a well-known inhibitor of biogas processes (specifically by inhibiting methanogens) [40], and this could possibly have caused the VFA accumulation followed by foaming. However, taking into account the pH (7.6–7.7) and temperature (37 °C), the level of free ammonia was calculated and found to be at most only around 0.16 g/L. This level was still low and below levels previously shown to cause inhibition [41]. Thus, a more likely explanation for the foaming was the introduction of starch by the MFV addition, which is typically converted rapidly to VFAs [39]. Previous studies investigating the effect of feeding regime on reactor performance have reported somewhat contradictory results and no consistent influence on key process parameters such as gas yield, degree of degradation, and VFA levels [9,19,21,22,23,24]. For example, no clear effect of the feeding regimes (feed every 2 days compared with daily) was seen on VFA, ammonia level and methane yield [19], while higher levels of VFA have been reported when feeding every 2 days compared with every 2 h [9] and every 6 h [21]. This inconsistency in results regarding the effect of feeding regime can be explained by differences in type of substrate, OLR, and feeding frequencies, with 2–48 h between feedings. Still, Mulat et al. (2016) and Ziels et al. (2018) obtained slightly higher methane yield (14 and 20%, respectively) with a less frequent feeding regime [9,22].

In conclusion, only small differences were observed between reactors with differences in feeding regimes in the present study. However, there were differences between the GB and GC reactors, with significantly higher specific methane production for GC reactors in the period after day 182 (student *t*-test *p* < 0.01) (Figure S2). This suggests that reactor performance was influenced by the original inoculum used for the start-up of the reactors in our previous study, where GB reactors produced significantly less methane than GC reactors in the start-up phase (within 1 HRT) [15]. The poor performance of GB reactors in our previous study was attributed to a higher ammonium-nitrogen level in the inoculum used for the start-up of these reactors [15]. In the present study the ammonium-nitrogen level increased, but to the same level in all reactors (GB: from 1.1 to 2.5 g/L, GC: from 1.0 to 2.5 g/L). Thus, a more likely explanation for the differing results obtained for GB and GC reactors is differences in the microbial community rather than the ammonia level per se, as discussed below.

### 3.3. Residual Methane Potential

For a production plant, the volumetric yield is highly important and thus continuously logged, while the specific yield (SMP) less often is considered. Thus, a decrease in degradation efficiency caused by a new cosubstrate, as shown in this study, can be somewhat hidden. Unfortunately, decreased degradation efficiency might increase the risk of methane emissions during storage of the digestate, as shown in several other studies [4,37,42]. To evaluate this risk, the residual methane production (RMP) was measured by incubation of digestate taken from all reactors before and after MFW addition (day 0 and day 231, respectively). The evaluation showed similar values for all reactors before MFW addition, i.e., 71 ± 5 mL CH_4_/g VS, on average for reactors GB0_0 and GC0_0 (pairwise *t*-test, *p* > 0.5) (Figure S3). However, after operation with MFW, the RMP was significantly higher showing on average 134 ± 12 mL CH_4_/g VS (pairwise *t*-test, *p* < 0.01), but with no significant differences between the reactors (Figure S3). Thus, MFW addition clearly increased the risk of methane emissions during storage, which was consistent with the decrease in the degree of degradation seen in the reactors and in the BMP tests.

### 3.4. Microbial Communities

#### 3.4.1. Diversity Indices

After quality trim and chimera check, 3,311,869 sequences (from 15,874 to 116,439 per sample) were retained. The triplicate samples were merged *in silico* and then subsampled based on the detected lowest sequences of the sample (41,100 sequences per sample). The number of observed OTUs across samples obtained from the rarefaction curve varied from 958 to 1666, with the lowest values for the GC reactors at the end of the experiment. At the start of the experiment, there was no significant difference in Chao1, Shannon, and Simpson indices of the observed OTUs between all four reactors (Table 3). However, the indices varied over time (Table 3) and the values appeared to fluctuate consistently with methane production and VFA levels in all four semi-continuous reactors (Table 3). Addition of MFW appeared to cause an overall decrease in diversity compared with operation with only the grass–manure mixture and this decrease was independent of the feeding regime.

Several previous studies have shown a correlation between high methane production and high diversity of microbial community, especially when the process was operated with complex substrates or in fluctuating process conditions, suggesting that a more diverse microbial community allows activation of multiple metabolic pathways and consequently high methane production [14,15,43]. However, under constant conditions, a specialized community can be expected to be more efficient [21]. In the present study, the GC reactors showed significantly higher methane production than GB reactors at the end of the experiment, displayed the greatest decrease in OTUs richness and in the Simpson and Shannon indices.

Previous studies have also shown that microbial community diversity can be affected by different feeding regimes. Digesters fed with lower frequency (every 2 days compared with daily or every 2 h) have been shown to form a more diverse microbial community [9,19]. In line with this, GB1 and GC1, receiving the MFW all at once, showed a slightly higher average number of observed OTUs and Shannon index, respectively, than GB2 and GC2, but this difference was not statistically significant (Table 3).

#### 3.4.2. Phylogenetic Analysis

**Figure 2:**
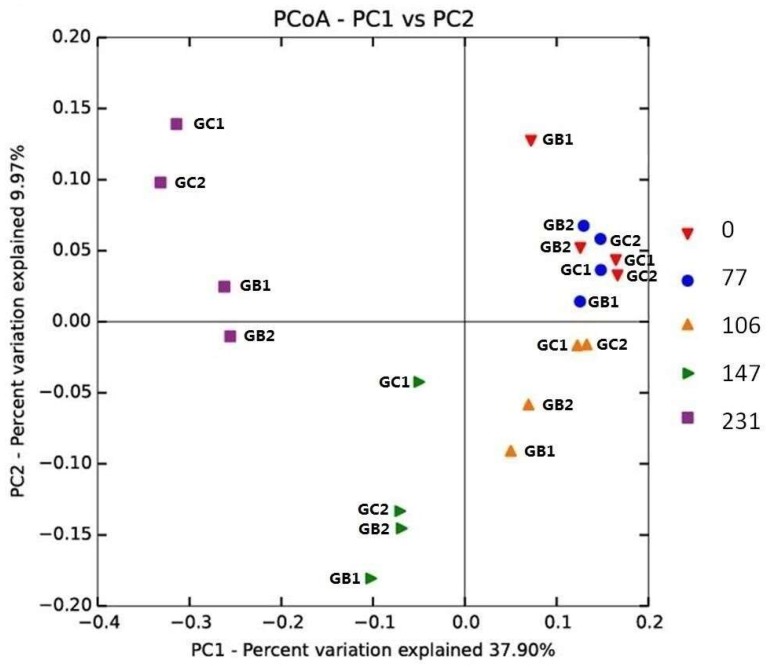
Phylogenetic distance between samples as determined by unweighted UniFrac principal coordinate analysis (PCoA). Sample legend arranged by time (day 0, 77, 106, 147, and 231).

The microbial community composition, analyzed by an unweighted UniFrac principal coordinate analysis (PCoA), was similar at the beginning of the experiment. However, the community changed over time and at the end (day 231) a separation was seen between GC and GB reactors, suggesting the importance of the original inoculum (Figure 2). For the different feeding regimes, however, no clear separation between GB1, GB2 and GC1, GC2 was observed (Figure 2).

Irrespective of MFW addition, the phyla Bacteroidetes (67.4 ± 12.7%) and Firmicutes (24.4 ± 9.7%) dominated in all processes and at all-time points, followed by the phylum Actinobacteria (2.2 ± 1.8%) (Figure S4). The phyla Tenericutes, Verrucomicrobia, Synergistetes, WWE1, and Proteobacteria were also detected in all reactors, but in relatively low abundance (<1%) (Figure S4). This dominance of the phyla Bacteroidetes and Firmicutes has been seen in various anaerobic digesters in many previous studies [15,44,45]. Members of these two phyla can utilize a broad range of organic compounds and are involved in the hydrolysis, fermentation, and acetogenesis steps of anaerobic digestion [2,46].

**Figure 3:**
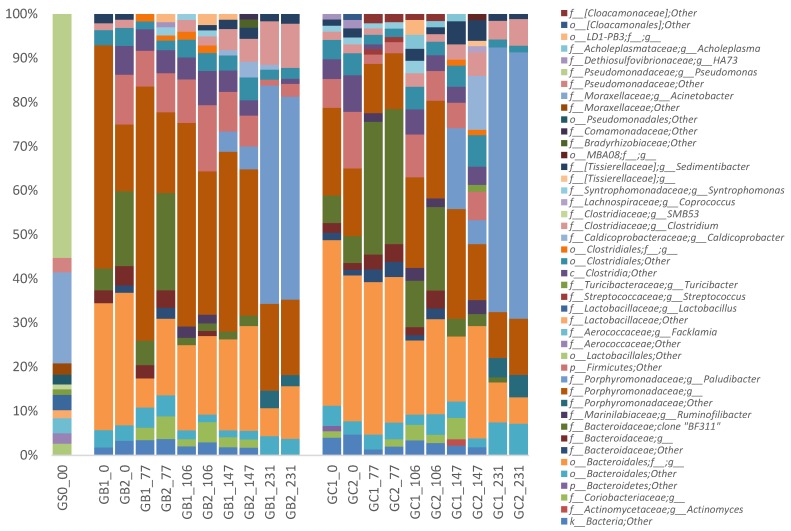
Relative abundance of bacterial 16S rRNA gene at genus level in the CSTR reactors (GB1, GB2, GC1, and GC2), arranged by time (day 0, 77, 106, 147, and 231 of operation) and the substrate sample (GS0_0). Relative abundance <1% were filtered out. The genus names were represented as the first letter of the closest classified taxonomical level plus the taxonomic name.

While no significant differences were seen on phylum level, MFW addition, independent of feeding regime, resulted in a similar shift in the overall microbial community pattern in all reactors at lower taxonomic level. The most pronounced change was an increase in the relative abundance of the genus *Paludibacter* (family Paludibacteraceae, order Bacteroidales, phylum Bacteroidetes) from <0.1% (day 0) to an average of 49.9 ± 7.5% (day 231) (Figure 3). This increase was seen in all reactors but was more pronounced in GC1 and GC2 (on average 12.7% higher than in GB1 and GB2). This difference was most likely the cause of the separation in the PCoA analysis at the last time point (i.e., day 231) (Figure 2). The genus *Paludibacter* was also found in the grass–manure mixture and in the original inocula for GB and GC, as described in our previous study [15], but here only at very low relative abundance (<0.1%). The genus *Paludibacter* is strictly anaerobic and can utilize various sugars such as arabinose, xylose, cellobiose, fructose, galactose, glucose, mannose, maltose, melibiose, glycogen, and soluble starch while producing acetate and propionate as major fermentation end-products [47]. Thus, in this study, the high level of starch in MFW probably enhanced the growth of this genus. Members of this genus have been found at various relative abundances in other anaerobic digesters and also in other anaerobic environments, such as cow manure, wetlands, sludge from alkali-hydrolyzed rice straw, and plant residues in irrigated rice-field soil [47,48,49,50,51,52]. The genus *Paludibacter* was recently also suggested as a potential cellulose degrader [51,53].

With the increase in *Paludibacter*, the average relative abundance of an uncultured rumen bacterium clone BF311 (belonging to unclassified order Bacteroidales) gradually decreased in all reactors after the addition of MFW, from 20.0 ± 10.4% (day 77) to 0.9 ± 0.2% (day 231), but with no significant difference between reactors (Figure 3). This uncultured rumen bacterium clone BF311 (GenBank: EU850525.1) is one partial sequence of 16S ribosomal RNA genes from a series of clones made by Satitmanwiwat et al. (2008, Thailand) (https://www.ncbi.nlm.nih.gov/nuccore/197940871). However, it was mistakenly assigned as genus *BF311* in the Greengene database and thus wrongly cited by other studies [54,55,56]. Still, BF311 has been reported in cattle rumen and horse feces samples [54,55]. However, to our knowledge no previous publication other than ours has found BF311 in biogas digesters. In our previous study, the relative abundance increased differently in GB and GC reactors, from 0.5% to 15.1% and from 2.5% to 5.2%, respectively, when operated with the same grass–manure mixture as used in the present study for over 3 HRT (i.e., 154 days) [15]. BF311 has been suggested to play an important role in lignocellulose degradation in rumen environments [55,56]. In this study, BF311 was possibly outcompeted by representatives from the genus *Paludibacter*.

Class Clostridia (phylum Firmicutes) also slightly decreased in response to MFW addition in all reactors, from average levels of 17.2 ± 4.2% to 10.6 ± 1.9%. However, the levels increased again around day 146 (to on average 26.0 ± 8.0%), i.e., in the period of VFA accumulation and slight reactor instability. During reactor recovery, the levels again decreased, but to different levels in the different reactors, ranging from 18.5% in GB reactors to 9.6% in GC. These changes in the class Clostridia were mainly caused by two unclassified families and the genus *Caldicoprobacter* (family Caldicoprobacteraceae) (Figure 3). Members of this genus can utilize various sugars, but also xylan and pyruvate, and produce acetate, lactate, and hydrogen as end-products [57,58]. The genus *Caldicoprobacter* has also been found to be enriched in anaerobic digesters fed lignocellulosic biomass under both mesophilic and thermophilic conditions [59,60]. Moreover, it has been shown to dominate in an anaerobic digester with high total ammonium-nitrogen (5 to 25 g/L) and, as in this study, high VFA levels (>4 g/L) [61].

Moreover, a slight increase in the genus *Clostridium* (family Clostridiaceae, phylum Firmicutes) from 1.6 ± 0.2% (day 0) to 6.9 ± 2.6% (day 231) (Figure 3), irrespective of the total changes in the level of Class Clostridia, was observed after MFW addition in all reactors. This genus contains organisms active both during fermentation and anaerobic oxidation that can utilize proteins and carbohydrates, and their corresponding monomers, while producing different fatty acids as end-products of their metabolism [62,63,64]. This increase is probably directly related to MFW addition and the observed increased in VFA level at the same time point [65]. A slight increase in relative abundance of the phylum Actinobacteria (mostly contributed by the family Coriobacteriaceae), from 1.2 ± 0.6% to 3.1 ± 2.1%, was also seen at the time of VFA accumulation and foaming (day 146). This phylum contains many acid-producing bacteria and has previously been found to increase in the deteriorative phase of an anaerobic process [44]. The family Coriobacteriaceae has been shown to dominate in an anaerobic digester operating with wastewater sludge and is suggested to convert lignocellulose hydrolysates into lactic acid and acetic acid [66,67].

Among the Archaea, the phyla Euryarchaeota and Crenarchaeota dominated (Figure S4). However, as seen in several other studies of biogas digesters [15,68,69], the total relative abundance of Archaea was very low, in this study less than 0.3% across all samples (Figure S4). Thus based on the sequencing results it was difficult to reveal detailed information about the methanogenic community. Still, order Methanobacteriales, Methanomicrobiales, and Methanosarcinales, with the dominance of order Methanobacteriales and Methanosarcinales, was detected in all four reactors before (day 0) and after (day 231) the MFW addition (Figure S5). These three methanogenic groups are commonly found in various anaerobic digesters [8,27], and members within these orders can all utilize hydrogen [8]. Members of Methanosarcinales can in addition utilize methanol and acetate for methane formation [8]. qPCR analysis furthermore revealed that all four reactors had a similar average gene abundance of these three methanogenic groups at the beginning (day 0) but at the end of the experiment (day 231) a significantly lower abundance, compared to the starting point, was seen for all groups (student *t*-test, *p* < 0.01) (Figure S6). However, a difference was observed in the reactors as a higher abundance of all three methanogenic groups was seen in GC compared to GB reactors (Figure S6), which could also contribute the separation of GB and GC at the day 231 in the PCoA plot (Figure 2). Possibly the higher abundance of methanogens in reactor GC could also explain the slightly higher methane production in these reactors. However, no clear difference could be observed for the different feeding regimes.

The different feeding regime showed no clear effect on the overall microbial community in this study. Similar results were obtained in a previous study during operation of CSTRs fed with glucose (once and twice a day and every 2 days) [23]. In contrast, a slight increase in microbial community richness was observed in a study using a starch-rich synthetic substrate fed every two days compared with daily feedings [19]. This is consistent with findings in the present study of higher richness in GB1 and GC1 compared with GB2 and GB2. Similarly, previous studies evaluating different feeding regimes have found effects of certain microbial groups. For example, during co-digestion of manure and oleate, the community fraction of the genus *Syntrophomonas* was higher when the oleate was fed every 2 days compared with every 6 h [22]. However, the interactions between feeding regime, digester performance (including methane production and process parameters), and microbial community remain somewhat unclear, as various feeding regimes have been shown to cause changes in microbial dynamics without affecting digester performance and vice versa [9,19,21,70].

#### 3.4.3. T-RFLP

In our previous study, the T-RFLP profiles for glycoside hydrolase families 5 (*cel5*) and 48 (*cel48*) genes differed between the original inocula used to start the GB and GC reactors [15]. After 3 HRT of operation with the grass–manure mixture, the various T-RFLP profiles became more similar and the community in both GB and GC reactors and the *cel5* and *cel 48* profile were dominated by T-RFs 74, 222, 228bp, and T-RF 328bp, respectively, according to clone libraries represented by *Clostridium cellulovorans* (WP_010075948, 60.7% identity), *Prevotella buccae* (WP_004346180, 55.1% identity), *Bacteroides uniformis* (WP_061411411, 67.5%, identity), and *Herbinix* sp. *SD1D* (WP_058258585, 89.7% identity), respectively [15].

**Figure 4:**
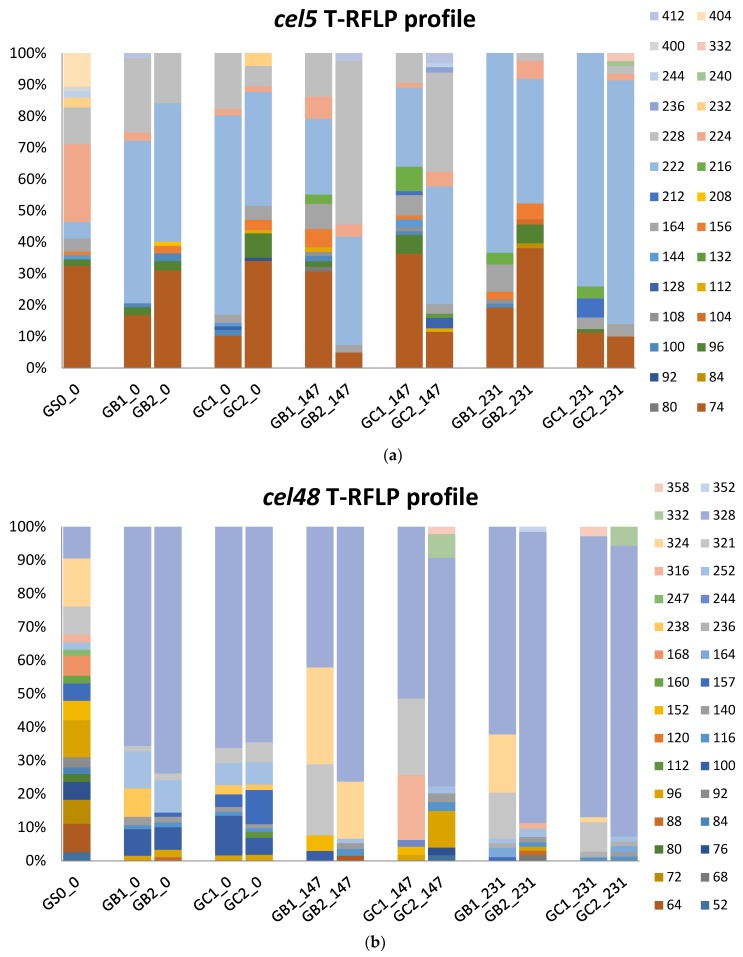
T-RFLP profile representing the community of glycoside hydrolase gene family 5 (*cel5*) (**a**) and 48 (*cel48*) (**b**) in the reactor samples (GB1, GB2, GC1, and GC2), arranged by time (day 0, 147, and 231) and the substrate sample (GS0_0).

In the present study, the addition of MFW as a cosubstrate changed both the *cel5* and *cel48* communities, not significantly in composition but somewhat more in relative abundance (Figure 4). For the *cel5* community, T-RF 222bp became slightly more abundant across all reactor samples by the end of the experiment (from 48.8 ± 11.6% to 56.8 ± 21.9%), while T-RF 228bp decreased from 15.9 ± 7.2% to 1.2 ± 1.4%. T-RF 74bp showed no significant trend in response to MFW addition (Figure 4a). For the *cel 48* community, T-RF 328bp increased in all reactor samples, from 67.5 ± 4.6% to 80.1 ± 13.7% (Figure 4b). The four bacteria representing these dominant T-RFs have been found in various anaerobic environments and show potential lignocellulolytic capacity [71,72,73,74]. *Prevotella buccae, Bacteroides uniformis*, and *Herbinix* sp. *SD1D* have also been shown able to utilize starch [75,76,77], most likely explaining the enrichment induced by MFW addition in the present study.

A different pattern in the T-RFLP profile was also seen in response to the feeding regime, with T-RF 216bp (4.6 ± 2.1%, *cel5*, not identified) and T-RF 321bp (16.7 ± 6.6%, *cel48*) mainly detected in samples where all MFW and the grass–manure mixture were fed simultaneously (Figure 4). T-RF 321bp has previously been shown to correspond to a clone most closely related to *Clostridium thermocellum* (ACT46162), with 75% identity [12]. This bacterium is reported to be a highly potent cellulose degrader and to be enriched in anaerobic digesters fed lignocellulose-rich materials [12,15,78]. Moreover, a species of this bacterium is reported to be capable of producing an extracellular amylase when grown on starch [79]. The higher abundance of this bacterium possibly explains the higher degradation efficiency of cellulose seen in GB1 and GC1 compared with GB2 and GC2 in BMP test II.

## 4. Conclusions

Addition of MFW to four semi-continuous processes that had been operated with a grass-manure mixture for ~200 days, and showed similar performance and microbial community structure, resulted in a significant increase in volumetric methane production and a concomitant decrease in specific methane production and substrate degradation efficiency. The magnitude of the decrease varied between the processes and appeared to relate to the initial inoculum used for startup. This may have been caused by differences in the microbial community prevailing in the initial inoculum, suggesting that the original inoculum can profoundly influence biogas production performance in the long term and affect microbial responses to process operation changes, which confirmed our hypothesis. Applying different feeding regimes for MFW addition had no clear influence on methane production or overall microbial community structure, but had an impact on the development of the cellulose-degrading community. Adding the MFW load all at once rather than in two portions at 2-h intervals gave slightly higher cellulose conversion activity (as indicated by BMP tests), possibly caused by a higher abundance of *Clostridium thermocellum*.

## Figures and Tables

**Table 1:** Composition of the grass-manure mix and milled feed wheat (MFW) substrates. Values (%) based on wet weight.

	VS	Crude Protein	Starch	Crude Fat	Crude Fiber	Ash
Manure-grass mixture	10.2	2.1	0.4	4.9	26.2	9.0
MFW	84.0	16.0	28.0	5.0	6.5	3.5

**Table 2:** Final methane potential (mL CH_4_/g VS) and time taken to reach 100%, 80%, and 50% of this potential with cellulose, grass–manure mixture, and milled feed wheat (MFW) substrates for the two different inocula (GB, GC) in the biomethane potential (BMP) test. The methane values were standardized to normal atmospheric pressure (273.15 K, 1 bar).

		Cellulose				Grass-manure mixture				MFW			
		Days to Reach % of Final Potential			Final Potential	Days to Reach % of Final Potential			Final Potential	Days to Reach % of Final Potential			Final Potential
Test	Inoculum	100%	80%	50%		100%	80%	50%		100%	80%	50%	
I	GB0_0	28	14	9	261 ± 33	52	16	5	329 ± 27	28	14	4	282 ± 24
	GC0_0	28	14	9	281 ± 17	52	17	7	382 ± 71	28	13	5	307 ± 53
II	GB1_231	119	56	21	350 ± 43	47	17	9	252 ± 29	65	27	15	278 ± 13
	GB2_231	119	61	35	270 ± 72	119	55	8	289 ± 11	119	54	19	322 ± 51
	GC1_231	119	61	35	308 ± 4	119	45	17	327 ± 46	119	54	25	333 ± 43
	GC2_231	119	61	38	274 ± 30	119	37	15	379 ± 73	119	39	25	307 ± 14

**Table 3:** Summary of observed OTUs, Chao1, Shannon, and Simpson index values.

Sample	Chao1	Observed OTUs	Shannon	Simpson
GB1_0	1571	1302	6.178	0.950
GB2_0	1619	1439	6.473	0.953
GB1_77	1410	1159	5.291	0.898
GB2_77	1485	1260	6.054	0.938
GB1_106	1673	1364	5.749	0.921
GB2_106	1773	1435	6.254	0.946
GB1_147	1477	1232	6.181	0.943
GB2_147	1544	1291	5.982	0.935
GB1_231	1453	1104	5.410	0.887
GB2_231	1449	1085	5.365	0.893
GC1_0	1813	1664	7.210	0.959
GC2_0	1872	1666	7.119	0.963
GC1_77	1592	1299	5.583	0.891
GC2_77	1650	1386	5.979	0.910
GC1_106	1718	1529	7.073	0.970
GC2_106	1744	1509	6.748	0.955
GC1_147	1775	1448	6.681	0.962
GC2_147	1675	1439	7.202	0.972
GC1_231	1414	989	5.431	0.872
GC2_231	1444	958	5.054	0.831

## Supplementary Materials

The following are available online at http://www.mdpi.com/2076-2607/6/3/80/s1, Figure S1: Accumulated methane (CH_4_) production (mL/g VS) using digestate taken from the end of the CSTR test (GB1_231 and GB2_231), operating with cellulose as substrate, Figure S2: Specific average methane (CH_4_) production (mL/g VS day) of four continuous laboratory-scale biogas reactors originally started with two different types of inoculum (GB and GC) and codigested with substrates of grass–manure and milled feed wheat (MFW) in two feeding approaches at 37 °C, Figure S3: Residual methane (CH_4_) production (mL/g VS) of digestate taken from all reactors before (GB0_0 and GC0_0) and after (GB1_231, GB2_231, GC1_231, and GC2_231) addition of milled feed wheat (MFW), Figure S4: Relative abundance of bacterial 16S rRNA gene at phylum level in the CSTR samples (GB1, GB2, GC1, and GC2), arranged by time (day 0, 77, 106, 147, and 231) and the substrate sample (GS0_0), Figure S5: Relative abundance of Archaea 16S rRNA gene based on next-generation amplicon sequencing at order level in the CSTR samples (GB1, GB2, GC1, and GC2), arranged by time (day 0 and 231), Figure S6: Average log gene abundance per mL sample obtained in qPCR analysis of the main methanogenic populations in the CSTR samples (GB1, GB2, GC1, and GC2), arranged by time (day 0 and 231), Table S1: Total changes over time in volatile fatty acid concentration (VFA, g/L) (including acetate, propionate, I-butyrate, butyrate, I-valerate, and valerate) in the different reactors. Pro/Ac = propionate to acetate ratio.
