# Supplementary material for: Substrate-Induced Response in Biogas Process Performance and Microbial Community Relates Back to Inoculum Source
Source: Microorganisms. 2018 Aug 5;6(3):80. doi: 10.3390/microorganisms6030080 (PMC6163493; doi:10.3390/microorganisms6030080)
Supplement: Supplementary file 1 [file microorganisms-06-00080-s001.zip › Figure S1.docx]

Figure S1. Accumulated methane (CH_4_) production (mL/g VS) using digestate taken from the end of the CSTR test (GB1_231, GB2_231), operating with cellulose as substrate.
