# Supplementary material for: Substrate-Induced Response in Biogas Process Performance and Microbial Community Relates Back to Inoculum Source
Source: Microorganisms. 2018 Aug 5;6(3):80. doi: 10.3390/microorganisms6030080 (PMC6163493; doi:10.3390/microorganisms6030080)
Supplement: Supplementary file 1 [file microorganisms-06-00080-s001.zip › Figure S2.docx]

Figure S2. Specific average methane (CH_4_) production (mL/g VS day) of four continuous laboratory-scale biogas reactors originally started with two different types of inoculum (GB, GC) and co-digested with substrates of grass-manure and milled feed wheat (MFW) in two feeding approaches at 37 °C.
