# Supplementary material for: Substrate-Induced Response in Biogas Process Performance and Microbial Community Relates Back to Inoculum Source
Source: Microorganisms. 2018 Aug 5;6(3):80. doi: 10.3390/microorganisms6030080 (PMC6163493; doi:10.3390/microorganisms6030080)
Supplement: Supplementary file 1 [file microorganisms-06-00080-s001.zip › Figure S3.docx]

Figure S3. Residual methane (CH_4_) production (mL/g VS) of digestate taken from all reactors before (GB0_0 and GC0_0) and after (GB1_231, GB2_231, GC1_231, and GC2_231) addition of milled feed wheat (MFW).
