# Supplementary material for: Substrate-Induced Response in Biogas Process Performance and Microbial Community Relates Back to Inoculum Source
Source: Microorganisms. 2018 Aug 5;6(3):80. doi: 10.3390/microorganisms6030080 (PMC6163493; doi:10.3390/microorganisms6030080)
Supplement: Supplementary file 1 [file microorganisms-06-00080-s001.zip › Figure S4.docx]

Figure S4. Relative abundance of bacterial 16S rRNA gene at phylum level in the CSTR samples (GB1, GB2, GC1 and GC2), arranged by time (day 0, 77, 106, 147, and 231) and the substrate sample (GS0_0)
