# Supplementary material for: Substrate-Induced Response in Biogas Process Performance and Microbial Community Relates Back to Inoculum Source
Source: Microorganisms. 2018 Aug 5;6(3):80. doi: 10.3390/microorganisms6030080 (PMC6163493; doi:10.3390/microorganisms6030080)
Supplement: Supplementary file 1 [file microorganisms-06-00080-s001.zip › Figure S5.docx]

Figure S5. Relative abundance of Archaea 16S rRNA gene based on next-generation amplicon sequencingat order level in the CSTR samples (GB1, GB2, GC1 and GC2), arranged by time (day 0 and 231).
