# Supplementary material for: Substrate-Induced Response in Biogas Process Performance and Microbial Community Relates Back to Inoculum Source
Source: Microorganisms. 2018 Aug 5;6(3):80. doi: 10.3390/microorganisms6030080 (PMC6163493; doi:10.3390/microorganisms6030080)
Supplement: Supplementary file 1 [file microorganisms-06-00080-s001.zip › Figure S6.docx]

Figure S6. Average log gene abundance per mL sample obtained in qPCR analysis of the main methanogenic populations in the CSTR samples (GB1, GB2, GC1 and GC2), arranged by time (day 0 and 231).
