# Supplementary material for: Substrate-Induced Response in Biogas Process Performance and Microbial Community Relates Back to Inoculum Source
Source: Microorganisms. 2018 Aug 5;6(3):80. doi: 10.3390/microorganisms6030080 (PMC6163493; doi:10.3390/microorganisms6030080)
Supplement: Supplementary file 1 [file microorganisms-06-00080-s001.zip › Table S1.docx]

|  | GB1 |  |  | GB2 |  |  | GC1 |  |  | GC2 |  |  |
| --- | --- | --- | --- | --- | --- | --- | --- | --- | --- | --- | --- | --- |
| Days | Total VFA | Acetate | Pro/Ac | Total VFA | Acetate | Pro/Ac | Total VFA | Acetate | Pro/Ac | Total VFA | Acetate | Pro/Ac |
| 28 | 0.11 | 0.07 | 0.57 | 0.05 | 0.05 | 0.00 | 0.04 | 0.04 | 0.00 | 0.03 | 0.03 | 0.00 |
| 35 | 0.06 | 0.04 | 0.50 | 0.03 | 0.03 | 0.00 | 0.04 | 0.04 | 0.00 | 0.03 | 0.03 | 0.00 |
| 49 | 0.05 | 0.04 | 0.25 | 0.05 | 0.04 | 0.25 | 0.12 | 0.02 | 1.50 | 0.07 | 0.04 | 0.75 |
| 56 | 0.14 | 0.04 | 0.00 | 0.10 | 0.10 | 0.00 | 0.30 | 0.10 | 0.00 | 0.31 | 0.06 | 0.83 |
| 64 | 0.05 | 0.05 | 0.00 | 0.15 | 0.10 | 0.50 | 0.10 | 0.10 | 0.00 | 0.06 | 0.06 | 0.00 |
| 71 | 0.05 | 0.05 | 0.00 | 0.05 | 0.05 | 0.00 | 0.00 | 0.00 | 0.00 | 0.00 | 0.00 | 0.00 |
| 85 | 0.04 | 0.04 | 0.00 | 0.12 | 0.05 | 1.40 | 0.11 | 0.04 | 1.75 | 0.09 | 0.04 | 1.25 |
| 98 | 0.32 | 0.10 | 1.00 | 0.31 | 0.08 | 0.38 | 0.17 | 0.07 | 1.43 | 0.13 | 0.08 | 0.63 |
| 106 | 0.07 | 0.04 | 0.75 | 0.25 | 0.20 | 0.10 | 0.07 | 0.05 | 0.40 | 0.12 | 0.09 | 0.33 |
| 112 | 0.23 | 0.09 | 1.00 | 0.30 | 0.10 | 1.00 | 0.20 | 0.10 | 1.00 | 0.20 | 0.10 | 1.00 |
| 119 | 0.14 | 0.06 | 1.33 | 0.40 | 0.10 | 1.00 | 0.18 | 0.08 | 1.25 | 0.60 | 0.20 | 0.50 |
| 126 | 0.16 | 0.10 | 0.60 | 0.28 | 0.08 | 1.25 | 0.40 | 0.20 | 1.00 | 0.40 | 0.10 | 3.00 |
| 133 | 0.20 | 0.10 | 1.00 | 0.30 | 0.20 | 0.50 | 0.17 | 0.08 | 1.13 | 0.17 | 0.10 | 0.70 |
| 140 | 0.13 | 0.08 | 0.63 | 0.60 | 0.30 | 1.00 | 0.60 | 0.30 | 1.00 | 0.54 | 0.30 | 0.67 |
| 161 | 1.13 | 0.40 | 1.50 | 1.17 | 0.40 | 1.75 | 0.63 | 0.10 | 5.00 | 0.63 | 0.10 | 5.00 |
| 168 | 1.16 | 0.30 | 2.67 | 0.97 | 0.20 | 3.50 | 0.94 | 0.30 | 2.00 | 0.85 | 0.30 | 1.67 |
| 182 | 0.85 | 0.25 | 2.20 | 1.54 | 0.27 | 4.37 | 0.92 | 0.20 | 3.50 | 0.98 | 0.30 | 2.00 |
| 189 | 0.93 | 0.50 | 0.80 | 1.50 | 0.50 | 1.80 | 1.00 | 0.40 | 1.25 | 0.85 | 0.30 | 1.67 |
| 196 | 1.00 | 0.30 | 2.33 | 1.57 | 0.30 | 3.67 | 0.94 | 0.20 | 3.50 | 1.10 | 0.30 | 2.67 |
| 203 | 1.00 | 0.30 | 2.33 | 1.24 | 0.10 | 11.00 | 0.90 | 0.30 | 2.00 | 0.93 | 0.20 | 3.50 |
| 210 | 1.10 | 0.20 | 4.50 | 2.07 | 0.40 | 3.25 | 1.05 | 0.20 | 4.00 | 1.75 | 0.40 | 2.75 |
| 217 | 2.39 | 0.40 | 4.50 | 1.65 | 0.30 | 4.00 | 1.25 | 0.30 | 3.00 | 0.11 | 0.07 | 0.57 |
| 224 | 1.62 | 0.20 | 6.50 | 4.55 | 2.50 | 0.76 | 1.57 | 0.20 | 6.50 | 2.84 | 1.80 | 0.56 |
| 231 | 1.20 | 0.30 | 3.00 | 1.84 | 0.40 | 3.25 | 1.08 | 0.20 | 4.00 | 1.00 | 0.30 | 2.33 |

**Table S1.** Total changes over time in volatile fatty acid concentration (VFA, g/L) (including acetate, propionate, I-butyrate, butyrate, I-valerate, and valerate) in the different reactors. Pro/Ac = propionate to acetate ratio.
